# Atrial Fibrillation During Zanubrutinib Treatment in Chronic Lymphocytic Leukemia With a Historical Ibrutinib Control

**DOI:** 10.1002/jha2.70451

**Published:** 2026-11-14

**Authors:** Andrea Visentin, Enrica Antonia Martino, Alessandro Cellini, Francesco Angotzi, Annalisa Pitino, Andrea Duminuco, Ernesto Vigna, Raffaella Pasquale, Isacco Ferrarini, Riccardo Moia, Alessandro Sanna, Marina Motta, Massimo Moratti, Paolo Sportoletti, Annalisa Chiarenza, Alessandro Maggi, Valentina Zammit, Michele Merli, Idanna Innocenti, Claudia Giordano, Laura Nocilli, Massimiliano Postorino, Caterina Stelitano, Andrea Ferrario, Anna Maria Frustaci, Marcello Riva, Sara Pepe, Adalberto Ibatici, Stefania Scardino, Paola Anticoli Borza, Laura Ballotta, Salvatrice Mancuso, Francesco Malaspina, Anna Mele, Sara Galimberti, Gioacchino Catania, Annamaria Giordano, Ilaria Angeletti, Luana Schiattone, Elsa Pennese, Rosanna Miccolis, Angelo Fama, Giulio Giordano, Catello Califano, Antonella Bruzzese, Giuliana Farina, Pietro Bulian, Giacomo Loseto, Barbara Pocali, Vanessa Innao, Piero Galieni, Vincenzo Fraticelli, Candida Vitale, Azzurra Romeo, Marco Rossi, Ilaria Scortechini, Federico Vozella, Luigi Malandruccolo, Marzia Varettoni, Lucia Morello, Giuseppe Pietrantuono, Esmeralda Conte, Martina Cantelli, Roberta Murru, Daniele Caracciolo, Enrico Derenzini, Valentina Di Martina, Roberto Marasca, Maria Ilaria Del Principe, Amalia Figuera, Marta Coscia, Nicola Di Renzo, Luca Laurenti, Nicola Amodio, Alessandro Allegra, Vittorio Del Fabro, Paolo Fiumara, Luca Scalise, Fabio Stagno, Giorgio Tona, Pellegrino Musto, Laura Caruso, Arcangelo Liso, Alessandra Tedeschi, Gianluca Gaidano, Francesca Romana Mauro, Giovanni Tripepi, Fortunato Morabito, Giuseppe Alberto Palumbo, Francesco Di Raimondo, Valter Gattei, Livio Trentin, Massimo Gentile

**Affiliations:** ^1^ Hematology Unit Department of Medicine‐DIMED University of Padova Padova Italy; ^2^ Hematology Unit Department of Onco‐Hematology Azienda Ospedaliera Annunziata Cosenza Italy; ^3^ Institute of Clinical Physiology (IFC‐CNR) Rome Italy; ^4^ Hematology and Stem Cell Transplantation Unit A.O.U.Policlinico G. Rodolico – San Marco Catania Italy; ^5^ SOC Clinica Ematologia Azienda Sanitaria Universitaria Friuli Centrale (ASU FC) Udine Italy; ^6^ Department of Engineering for Innovation Medicine University of Verona Verona Italy; ^7^ Division of Hematology Department of Translational Medicine Università del Piemonte Orientale Novara Italy; ^8^ Hematology Unit Deparment of Oncology AOU‐Careggi Florence Italy; ^9^ S.C. Ematologia ASST – Spedali Civili di Brescia Brescia Italy; ^10^ Centro di Ricerca Emato‐Oncologica (CREO) Dipartimento Di Medicina e Chirurgia Università di Perugia Perugia Italy; ^11^ Haematology Ospedale G. Moscati Taranto Italy; ^12^ Oncoematology and BMT Unit Oncology Department Ospedale La maddalena Palermo Palermo Italy; ^13^ Hematology Ospedale Maggiore Policlinico Fondazione IRCCS Ca' Granda Milano Italy; ^14^ Area Ematologia Dipartimento Di Scienze di Laboratorio ed Ematologiche Presso Fondazione Policlinico Universitario A. Gemelli IRCCS Roma Italy; ^15^ Department of Clinical Medicine and Surgery Federico II University Medical School Naples Italy; ^16^ Department of Hematology Azienda Ospedaliera Papardo Messina Italy; ^17^ Sant'Eugenio Hospital AslRoma2 Department of Biomedicine and Prevention Tor Vergata University of Rome Rome Italy; ^18^ Department of Hematology Azienda Ospedaliera Bianchi Melacrino Morelli Reggio Calabria Italy; ^19^ SC Ematologia Ospedale di Circolo di Varese ASST dei Sette Laghi Varese Italy; ^20^ Department of Haematology Niguarda Cancer Center ASST Grande Ospedale Metropolitano Niguarda Milan Italy; ^21^ Hematology Department Ospedale San Bortolo di Vicenza Vicenza Italy; ^22^ Hematology Department of Translational and Precision Medicine Sapienza University Rome Italy; ^23^ Hematology and Cellular Therapy Unit IRCCS Ospedale Policlinico San Martino Genoa Italy; ^24^ Hematology and Stem Cell Transplant Unit "Vito Fazzi" Hospital Lecce Italy; ^25^ UOC Ematologia Azienda Ospedaliera San Giovanni‐Addolorata Rome Italy; ^26^ Hematology Unit ASUGI (Azienda Sanitaria Universitaria Giuliano Isontina) Ospedale Maggiore Trieste Trieste Italy; ^27^ Hematology Unit Department of Health Promotion, Mother and Child Care, Internal Medicine and Medical Specialties (PROMISE) University of Palermo Palermo Italy; ^28^ Hematology Unit IRCCS, IRST Istituto Scientifico Romagnolo per Lo Studio e la Cura dei Tumori "Dino Amadori Meldola Italy; ^29^ Haematology Ospedale Cardinale Panico Tricase (Lecce) Italy; ^30^ Division of Hematology University Hospital of Pisa Pisa Italy; ^31^ SCDU Ematologia IRCCS AOU SS Antonio e Biagio e Cesare Arrigo Alessandria Italy; ^32^ Unit of Hematology and Stem Cell Transplantation AOUC Policlinico Bari Italy; ^33^ Hematology/Oncology Unit Terni Hospital Department of Medicine and Surgery University of Perugia Terni Italy; ^34^ Hematology Unit S. Spirito Hospital Pescara Italy; ^35^ Department of Hematology ASL BT Barletta Italy; ^36^ Hematology Unit Department of Internal Medicine Ospedale G. Mazzini Teramo Italy; ^37^ Medicina Servizio e Ambulatorio di Ematologia Unità Operativa Complessa Ospedale di Riferimento Regionale Antonio Cardarelli Campobasso Italy; ^38^ Hematology Unit "A. Tortora" Hospital Pagani Italy; ^39^ Hematology Unit Department of Oncohematology AORN San Anna e San Sebastiano Caserta Italy; ^40^ Clinical and Experimental Onco‐Hematology Unit Centro di Riferimento Oncologico di Aviano (CRO) IRCCS Aviano Italy; ^41^ Hematology and Cell Therapy Unit IRCCS Istituto Tumori 'Giovanni Paolo II' Bari Italy; ^42^ Division of Hematology and Stem Cell Transplantation Unit Cardarelli Hospital Naples Italy; ^43^ Hematology Unit Azienda Ospedaliera di Rilievo Nazionale e di Alta Specializzazione (ARNAS) Garibaldi Catania Italy; ^44^ Department of Haematology and Stem Cell Transplantation Unit C. e G. Mazzoni Hospital Ascoli Piceno Italy; ^45^ Oncohematology Unit Department of Oncology Responsible Research Hospital Campobasso Italy; ^46^ Division of Hematology A.O.U. Città della Salute e della Scienza di Torino Torino Italy; ^47^ Department of Molecular Biotechnology and Health Sciences University of Turin Turin Italy; ^48^ Hematology Unit Ospedale Santa Maria Goretti Latina Italy; ^49^ Department of Hematology‐Oncology Azienda Universitaria Ospedaliera Renato Dulbecco Catanzaro Italy; ^50^ Department of Experimental and Clinical Medicine Magna Grecia University Catanzaro Italy; ^51^ Azienda Ospedaliero Universitaria delle Marche Ancona Italy; ^52^ Ematologia e Trapianto Di Cellule Staminali Fondazione Policlinico Universitario Campus Bio‐Medico Roma Italy; ^53^ Hematology Unit Ospedale "Fabrizio Spaziani Frosinone Italy; ^54^ SC Ematologia 1 Fondazione IRCCS Policlinico San Matteo Pavia Italy; ^55^ Unità Operativa di Ematologia Ospedale Guglielmo da Saliceto Piacenza Italy; ^56^ Referral Cancer Center of Basilicata IRCCS CROB Rionero in Vulture Italy; ^57^ Haematology Unit Department of Clinical and Molecular Medicine Sant'Andrea University Hospital Sapienza University Rome Italy; ^58^ Unità di Ematologia Ospedale Santa Maria delle Croci Ravenna Italy; ^59^ Hematology and Stem Cell Transplantation Unit Ospedale Oncologico A. Businco ARNAS G. Brotzu Cagliari Italy; ^60^ Division of Onco‐Hematology Department of Health Sciences University of Milan (IEO) European Institute of Oncology IRCCS Milan Italy; ^61^ Hematology Unit Ospedale San Vincenzo Taormina Italy; ^62^ Hematology Unit Department of Oncology and Hematology Policlinico Modena Azienda‐Ospedaliero Universitaria (AOU) of Modena Modena Italy; ^63^ Hematology Unit Department of Human Pathology in Adulthood and Childhood "Gaetano Barresi University Hospital "G. Martino" Messina Messina Italy; ^64^ Faculty of Medicine and Surgery "Kore" University of Enna Enna Italy; ^65^ Division of Hematology Ospedale "Sant'elia Caltanissetta Italy; ^66^ Department of Precision and Regenerative Medicine and Ionian Area "Aldo Moro" University School of Medicine Bari Italy; ^67^ Clinical Epidemiology and Physiopathology of Renal Diseases and Hypertension of Reggio Calabria Institute of Clinical Physiology (IFC‐CNR) Reggio Calabria Italy; ^68^ Dafne srl Reggio Calabria Italy; ^69^ Department of Pharmacy, Health and Nutritional Science University of Calabria Rende Italy

**Keywords:** atrial fibrillation score, atrial fibrillation, cardiovascular risk, chronic lymphocytic leukemia, zanubrutinib

## Abstract

**Introduction:**

Atrial fibrillation (AF) complicates Bruton tyrosine kinase inhibitor therapy in chronic lymphocytic leukemia.

**Methods:**

We retrospectively evaluated 679 zanubrutinib‐treated patients across 52 Italian centers, with 354 historical ibrutinib‐treated controls. Shanafelt AF scores were assessed.

**Results:**

Nine zanubrutinib‐treated patients developed AF (1.33%) during median treatment of 15.5 months, versus 42 historical controls (11.9%) over median observation of 25 months. Five zanubrutinib events occurred among patients scoring ≥ 5. No affected patients experienced embolic or bleeding complications.

**Conclusion:**

AF was uncommon during zanubrutinib treatment, even in the higher‐risk subgroup. Historical comparisons remain limited, and exploratory score findings require confirmation with longer follow‐up.

**Trial Registration:**

The authors have confirmed clinical trial registration is not needed for this submission

1

Bruton tyrosine kinase inhibitors (BTKis) have revolutionized the treatment of chronic lymphocytic leukemia (CLL), becoming a cornerstone of therapy across both frontline and relapsed/refractory settings. Continuous BTKi therapy has translated into durable disease control and prolonged survival, leading to an increasing number of patients receiving these agents for extended periods. Alongside these remarkable therapeutic advances, however, cardiovascular adverse events have emerged as a major concern. Among them, atrial fibrillation (AF) represents one of the most frequent and clinically relevant toxicities, as it may increase the risk of thromboembolic complications, require anticoagulation, and ultimately may lead to treatment interruption or permanent discontinuation of BTKi therapy [1].

The incidence of AF differs among available BTKis, reflecting differences in kinase selectivity and off‐target effects. Ibrutinib, the first‐generation BTKi, has consistently been associated with the highest incidence of AF, whereas second‐generation BTKis, such as acalabrutinib and zanubrutinib, appear to confer a lower cardiovascular risk [2, 3]. Nevertheless, AF remains an important adverse event across the BTKi class, particularly in the elderly CLL population, which frequently presents with pre‐existing cardiovascular comorbidities [1]. Moreover, the reported incidence of AF varies substantially across randomized clinical trials and real‐world studies, likely owing to differences in patient characteristics, baseline cardiovascular risk, follow‐up duration, and methods of AF ascertainment [4, 5].

Given the clinical consequences of BTKi‐associated AF, accurate risk stratification before treatment initiation has become increasingly important. Several predictive models have been proposed to identify patients at increased risk of developing AF, but only a limited number have been specifically validated in patients with CLL [4, 5, 6, 7]. Among these, the Shanafelt Atrial Fibrillation Score (AFS) is one of the most widely studied and validated tools [6, 8]. The AFS incorporates readily available clinical variables that are independently associated with AF in patients with CLL, including advanced age (65–74 yrs: 2 points; > 74 yrs: 3 points), valvular heart disease (2 points), hypertension or male gender (1 point) [8]. Based on the cumulative score, patients are categorized into distinct risk groups, allowing estimation of their baseline likelihood of developing AF. Importantly, the AFS was subsequently evaluated in cohorts of patients treated with BTKi, demonstrating its ability to identify individuals at increased risk of treatment‐emergent AF [8]. In particular, patients with an AFS ≥ 5 consistently exhibited a substantially higher incidence of AF during treatment with both ibrutinib and acalabrutinib compared with those in lower‐risk categories [6, 8, 9].

The distribution of AF across AFS categories in contemporary real‐world populations treated with zanubrutinib remains incompletely characterized. We therefore examined AF events by baseline AFS category as an exploratory analysis.

We described AF during zanubrutinib treatment in the CLL‐ZANU2024 project, an Italian multicenter observational study collecting data on patients treated from October 2023 to May 2026 [10, 11], and retained the previously published ibrutinib cohort as a historical control [7]. This control comprised 354 patients (Table S1) managed in eight sites from 2012 to 2018, with 42 AF events and a median observation of 25 months. The cohorts were non‐contemporaneous. Comparisons are descriptive and unadjusted; differing observation periods and patient characteristics prevent estimation of a causal treatment effect. Both cohorts were stratified according to the Shanafelt AFS: age 65–74 years: 2 points; age ≥ 75 years: 3 points; valvular heart disease: 2 points; and hypertension and male sex, 1 point each [8].

The Zanubrutinib cohort comprised 679 patients treated at 52 Italian sites. Median age was 75.9 years (range 44–94.5), 61% were male, and hypertension was present in 38%; Type 2 diabetes mellitus and hypercholesterolemia were each present in 15% (Table 1). Twenty‐seven patients (4%) had a history of AF at baseline. During a median treatment duration of 15.5 months, nine patients had reported new‐onset AF, representing 1.33% of the 679 full cohort (Figure 1A), and 1.38% of at‐risk patient. AF occurred after a median exposure of 16 months. Five events occurred at age 65–74 years, three at ≥ 75 years, and one at < 65 years (Table S2). Four affected patients had a reported cardiovascular risk factor: hypertension in two, diabetes in one, and myocardial infarction in one. Four discontinued zanubrutinib and four temporarily interrupted treatment before restarting at 80 mg twice daily. Seven received beta‐blockers and one received flecainide. Eight received direct oral anticoagulants (seven apixaban and one edoxaban). None of the nine patients developed embolic or bleeding complications during the available follow‐up.

**TABLE 1:** Characteristics of the zanubrutinib cohort by reported new‐onset AF status.

	**Total population (*n* = 679)**	**Reported new‐onset AF (*n* = 9)**	**No reported new‐onset AF (*n* = 670)**
Age			
median years (range)	75.9 (44–94.5)	73.6 (47–81.9)	76 (44–94.5)
**Sex**, *n* (%)			
Male	413 (61%)	4	409
Female	266 (39%)	5	261
**Line of therapy**, *n* (%)			
Treatment naive	388 (57%)	5	383
Relapsed / refractory	291 (43%)	4	287
**Binet stage**, *n* (%)			
A	61 (9%)	0	61
B	254 (37%)	4	250
C	364 (54%)	5	359
**IGHV mutational status**, *n* (%)			
Mutated	202 (30%)	3	199
Germline	396 (58%)	6	390
Unknown	81 (12%)	0	81
**TP53 disruption**, *n* (%)			
Absent	414 (61%)	5	409
Present	182 (27%)	4	178
Unknown	83 (12%)	0	83
**Cardiovascular risk factor**, *n* (%)			
Hypertension	261 (38%)	2	259
Atrial fibrillation	27 (4%)	0	27
Hypercholesterolemia	100 (15%)	0	100
Congestive heart failure	27 (4%)	0	27
Peripheral arterial disease	7 (1%)	0	7
Cerebrovascular disease	36 (5%)	0	36
Type 2 diabetes mellitus	100 (15%)	1	99
Myocardial infarction	64 (9%)	1	63

*Note*: The full cohort includes 27 patients with baseline AF, all in the group without reported new‐onset AF. Counts are descriptive; the table is not restricted to the population at risk for first AF. Incident‐risk comparisons require exclusion of patients with prior AF. The historical ibrutinib control comprised 354 patients, including 42 with AF, whose characteristics are provided in Table S1.

Abbreviations: AF, atrial fibrillation; CLL, chronic lymphocytic leukemia.

**FIGURE 1:**
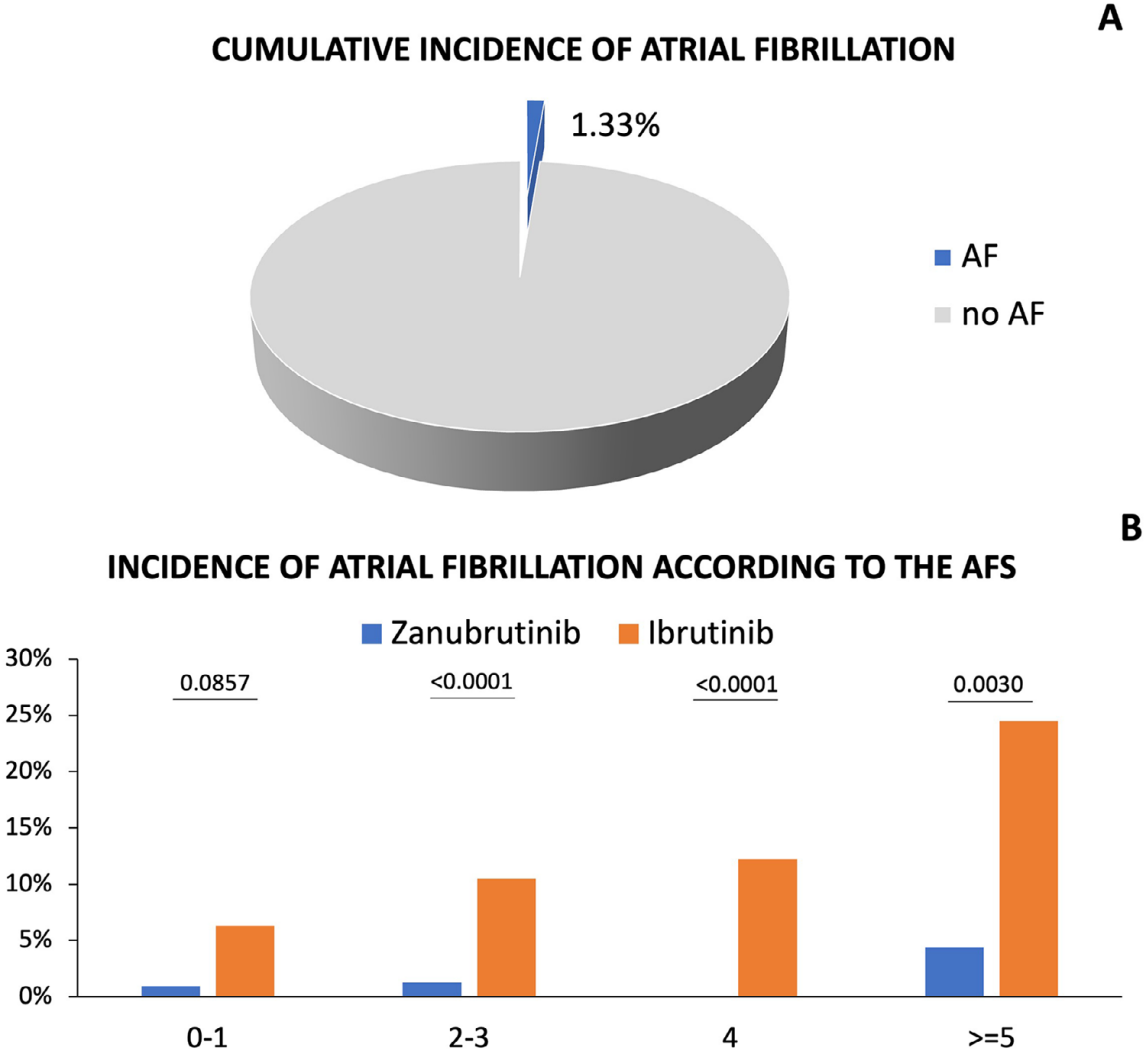
Reported AF during zanubrutinib treatment and historical ibrutinib comparison. (A) Crude proportion of the zanubrutinib cohort with reported new‐onset AF (9/679, 1.3%). (B) Comparison of crude overall AF proportions in the zanubrutinib cohort and historical ibrutinib control (42/354, 11.9%). Observation periods differ; these are unadjusted descriptive proportions, not time‐specific cumulative incidence estimates or causal treatment effects.

The AFS categories 0–1, 2–3, 4, and ≥ 5 included 105 (15.5%), 237 (34.9%), 224 (33.0%), and 113 (16.6%) patients, respectively. AF events in these categories numbered 1, 3, 0, and 5 (Table S2), corresponding to crude proportions of 1.0%, 1.3%, 0%, and 4.4% of the respective full‐cohort strata. These denominators include patients with baseline AF. Although five of the nine events occurred in the highest AFS category, the pattern was non‐monotonic, and the limited event count precludes validation of discrimination or calibration.

The crude AF proportion was numerically lower in the zanubrutinib cohort than in the historical ibrutinib control (9/679, 1.33%, vs. 42/354, 11.9%). The supplied age‐stratified historical summaries also show higher AF proportions with ibrutinib (Table S2). These observations are exploratory: median zanubrutinib treatment duration was 15.5 months, whereas median historical observation was 25 months, and the zanubrutinib cohort was older and included more treatment‐naive patients (57% vs. 25%). The estimate is immature because the median exposure at AF onset was 16 months. For context, randomized ALPINE data showed AF/flutter in 7.1% versus 17.0% with zanubrutinib and ibrutinib at median follow‐up of 42.5 months [2]. Our historical comparison does not replace this randomized evidence. The incidence of AF for zanubrutinib and ibrutinib is 1.03 (95% CI 0.49–2.03) and 6.15 (95% CI 4.43‐8.32) per 100 patient‐years, respectively.

Previous studies have examined AFS‐based risk stratification during ibrutinib and acalabrutinib treatment [6, 9]. Our historical patients treated with ibrutinib were re‐scored according to AFS in Tables S1 and S2. Compared to ibrutinib, zanubrutinib significantly reduced the risk of developing AF compared in patients with CLL aged 65–74 years (2.6% vs. 25.8%, *p* < 0.001) and ≥ 75 years (0.8% vs. 17%, *p* < 0.0001, Table S2). Of note, according to the AFS score, the risk is statistically decreased in patients with a score of 2–3 (1.3% vs. 10.5%, *p* < 0.0001), 4 points (0% vs. 12.2%, *p* < 0.0001), ≥ 5 points (4.6% vs. 24.5%, *p* = 0.003, Table S2, Figure 1B).

Baseline cardiovascular assessment and management of modifiable risk factors remain relevant during BTKi therapy [1, 12]. Limitations include the retrospective design, only nine zanubrutinib AF events, short observation, and use of a non‐contemporaneous historical control. Differences in centers, treatment practices, prior therapy, and AF ascertainment may introduce bias. Systematic rhythm monitoring was not routinely performed in the zanubrutinib cohort, potentially missing asymptomatic AF. Nevertheless, the large multicenter real‐world cohort and the consistency of our findings with prospective clinical studies strengthen the validity of our observations [13]. A low proportion of AF was observed during available zanubrutinib exposure, numerically below that in the historical ibrutinib control. Unequal observation and potential confounding limit interpretation. The concentration of events in the highest AFS category is exploratory and does not validate the score.

Overall, our data confirm zanubrutinib as a BTKi with an excellent cardiovascular safety profile [14] and demonstrate that the AFS might be a practical tool for identifying patients at relatively higher risk of AF, even in the era of second‐generation BTKis. Our results highlight that the AFS might be a simple and readily available tool for pretreatment cardiovascular risk stratification across different BTK inhibitors but require longer follow‐up and harmonized analyses.

## Author Contributions

A.V. designed the study, visited patients, and wrote the article. A.V., M.G., and E.A.M. analyzed data, visited patients, and revised the article. All the other authors revised the article, provided intellectual input, and visited patients.

## Funding

This study was supported by the PNRR‐MAD‐2022‐12375673.

## Ethics Statement

The study was approved by the ethics committees of participating institutions and conducted in accordance with the Declaration of Helsinki.

## Consent

Written informed consent has been obtained from the involved patients.

## Conflicts of Interest

Andrea Visentin and Massimo Gentile received research grants from BeOne Medicine and attended advisory boards organized by Abbvie, AstraZeneca, BeOne Medicine, Lilly, Johnson & Johnson, and Takeda. The other authors declare no conflicts of interest.

## Supporting information




**Supporting Information File 1**: jha270451‐sup‐0001‐Table S1.docx

## Data Availability

The data that support the findings of this study are available on request from the corresponding author. The data are not publicly available due to privacy or ethical restrictions.
