# Supplementary material for: Atrial Fibrillation During Zanubrutinib Treatment in Chronic Lymphocytic Leukemia With a Historical Ibrutinib Control
Source: EJHaem. 2026 Nov 14;7(6):e70451. doi: 10.1002/jha2.70451 (PMC13769379; doi:10.1002/jha2.70451)
Supplement: Supplementary file 1 [file JHA2-7-e70451-s001.docx]

**Table S1. Characteristics of the historical ibrutinib control (N=354)**

|  | **All**  **n=354** | **AF**  **n=42** | **No AF**  **n=312** |
| --- | --- | --- | --- |
| **Age (years)** |  |  |  |
| Median  Sta. dev. | 69.66  9.23 | 71.04  8.70 | 68.28  9.31 |
| **Gender** |  |  |  |
| Male  female | 226 (64%)  128 (36%) | 30 (71%)  12 (29%) | 196 (63%)  116 (37%) |
| **Therapy** |  |  |  |
| Treatment naive  Relapsed | 90 (25%)  264 (75%) | 13 (31%)  29 (69%) | 77 (25%)  235 (75%) |
| **TP53 abn** |  |  |  |
| normal  abnormal | 213 (60%)  135 (38%) | 23 (55%)  19 (45%) | 190 (62%)  116 (38%) |
| **IGHV status** |  |  |  |
| U-IGHV  M-IGHV | 216 (61%)  96 (27%) | 25 (66%)  13 (34%) | 191 (70%)  83 (30%) |
| **Valvular h.d.** |  |  |  |
| Yes  no | 34 (10%)  320 (90%) | 11 (26%)  31 (74%) | 23 (7%)  289 (93%) |
| **Cardiopathy** |  |  |  |
| Yes  no | 36 (10%)  318 (90%) | 11 (26%)  31 (74%) | 25 (8%)  287 (92%) |
| **Type 2 diabetes** |  |  |  |
| Yes  no | 37 (10%)  317 (90%) | 9 (21%)  33 (79%) | 28 (9%)  284 (91%) |

Missing data for IGHV 42, TP53 abnormalities 6.

**Table S2. Crude AF summaries by age and score category in zanubrutinib and historical ibrutinib cohorts**

| **Category** | **Zanubrutinib N** | **Zanubrutinib AF n (%)** | **p value** | **Historical ibrutinib N** | **Historical AF n (%)** | **p value** |
| --- | --- | --- | --- | --- | --- | --- |
| All patients | 679 | 9 (1.3%) | 0.1876 | 354 | 42 (11.9%) |  |
| Age <65 yr | 120 | 1 (0.8%) |  | 194 | 9 (4.6%) | 0.0955 |
| Age 65–74 yr | 192 | 5 (2.6%) |  | 66 | 17 (25.8%) | <0.0001 |
| Age ≥75 yr | 367 | 3 (0.8%) |  | 94 | 16 (17.0%) | <0.0001 |
| **AFS** |  |  |  |  |  |  |
| Score 0–1 | 105 | 1 (1.0%) | 0.0046 | 79 | 5 (6.3%) | 0.0857 |
| Score 2–3 | 237 | 3 (1.3%) |  | 152 | 16 (10.5%) | <0.0001 |
| Score 4 | 224 | 0 (0%) |  | 74 | 4 (12.2%) | <0.0001 |
| Score ≥5 | 113 | 5 (4.4%) |  | 49 | 12 (24.5%) | 0.0030 |

AF, atrial fibrillation. Zanubrutinib score strata use Shanafelt AFS.
